# Outcome of medical treatment of otogenic brain abcess without therapeutic drainage: A case of central vertigo

**DOI:** 10.1016/j.radcr.2022.06.102

**Published:** 2022-07-28

**Authors:** Soni Azhar Pribadi, Aan Dwi Prasetio, Putri Irsalina, Wardah Rahmatul Islamiyah, Paulus Sugianto

**Affiliations:** Department of Neurology, Faculty of Medicine, Airlangga University, Surabaya, Indonesia

**Keywords:** Otogenic brain abscess, Cerebellar abscess, Otitis media

## Abstract

Otogenic brain abscess is a severe infection that must be treated as early as possible. Rare cases with a high mortality rate can be reduced by recognizing the red flags of a brain abscess, such as headaches, mental status changes, fever, and focal neurological deficits. Those could be supported by modern diagnostic management and adequate antibiotic therapy that was able to penetrate the central nervous system and abscesses. We report a case of a cerebellar abscess of the 49-year-old man with the chief complaint of vertigo. It was accompanied by chronic progressive headache, fever, bidirectional nystagmus, abnormal Romberg test, and abnormal cerebellar signs. Magnetic resonance imaging (MRI) of the head with contrast showed a right cerebellar abscess with an infectious source of otitis media and mastoiditis. The MRI evaluation showed improvement after admistered metronidazole 500 mg every 6 hours (week 22) and cefixime 200 mg every 12 hours (week 13). Long-term antibiotic treatment can be an alternative if surgery cannot be performed. However, surgery is still considered if there is no good clinical response during medical therapy.

## Introduction

Central vertigo is divided into vascular and nonvascular causes, the most common being vertebrobasilar ischemic stroke. Among nonvascular reasons, cerebellopontine tumors such as acoustic neuroma are the most frequent and rare cases. In cases where central vertigo is chronic, headache accompanied by an ataxic gait may suggest a cerebellar abscess. Most abscesses that occur in the cerebellum are otogenic brain abscesses. Common symptoms of cerebellar abscess are gait ataxia and abnormal manifestations shown on cerebellar function tests. A cerebellar abscess is a rare case. The incidence of cerebellar abscess is estimated to be 0.3-0.9 per 100,000 population per year in developed countries, with a male-to-female ratio of 2:1 to 3:1 and an age range of 30-40 years. The frequency of cerebellar disturbances and vertigo is often underestimated due to inaccurate examination of the motor and vestibular systems. In acute vertigo, cerebellar lesions account for about two-thirds of central lesions, or about 2.5-6.5% of all critical cases. Morbidity and mortality rates are very high, but with advances in imaging and good antibiotic management, mortality rates can be reduced by 30%-50%. Inappropriate use of antibiotics can alter or hide the disease's characteristics, making diagnosis difficult. Morbidity and mortality rates are very high, but with advances in imaging and good antibiotic management, mortality rates can be reduced by 30%-50%. Inappropriate use of antibiotics can alter or hide the characteristics of the disease which can make diagnosis difficult [1].

Therefore, it is essential to fully understand the early symptoms and clinical manifestations and make proper diagnoses and treatments. Appropriate antibiotics and surgical intervention are crucial in treating otogenic brain abscesses. However, the extent of the surgical procedure and the optimal timing for this intervention is still a matter of debate. We report a patient with an otogenic brain abscess that developed central vertigo while treated with appropriate systemic antibiotic management.

## Case

**Fig. 1:**
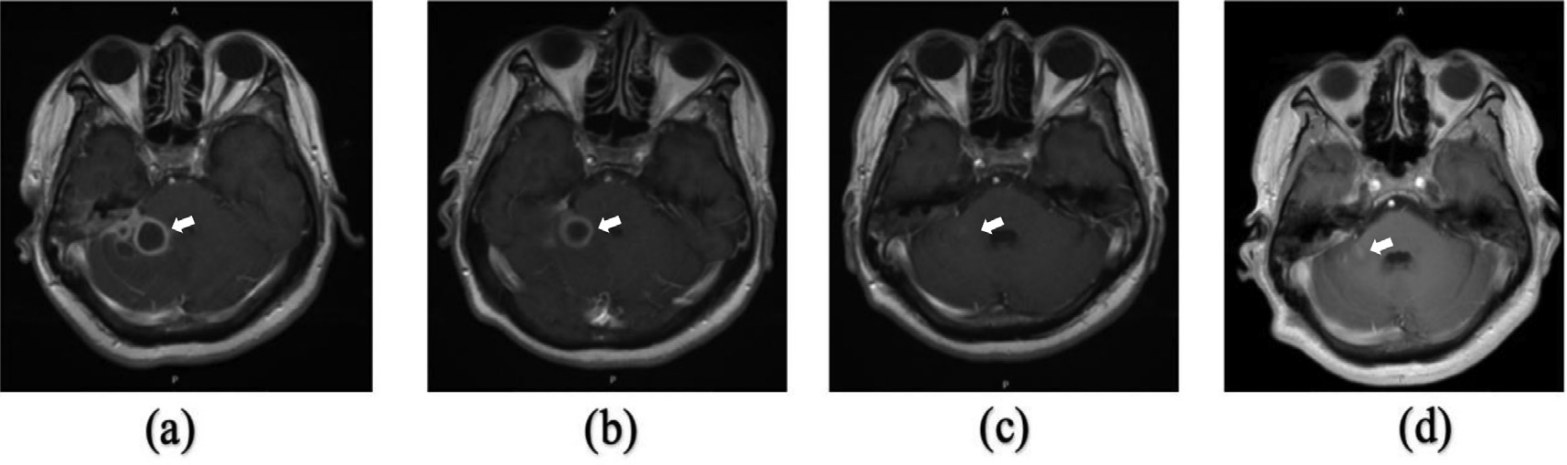
(a) MRI of the head with T1-weighted axial contrast before therapy. The lesion was seen pressing the right posterior aspect of the pons and the 4th ventricle medially, accompanied by leptomeningeal enhancement and right mastoiditis (arrow); (b) MRI of the head with T1-weighted axial contrast at week 5 showed the lesion appeared to be smaller; (c) T1-weighted axial contrast head MRI at week 22. The lesion showing contrast enhancement appeared to be shrinking; (d) 28th week T1-weighted axial contrast MRI of the head showing a relatively similar abscess size compared to week 22.

**Fig. 2:**
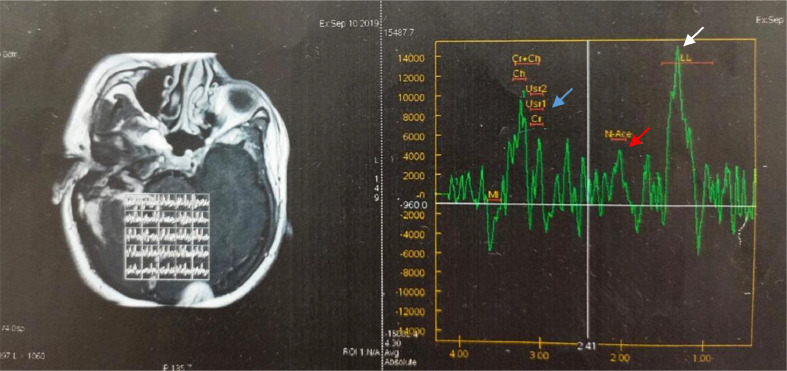
MR spectroscopy showed an increase in the lipid/lactate ratio (white arrow), no increase in the intra or perilesional choline/creatin (blue arrow) and choline/N-acetylaspartate (red arrow) ratios were seen.

**Fig. 3:**
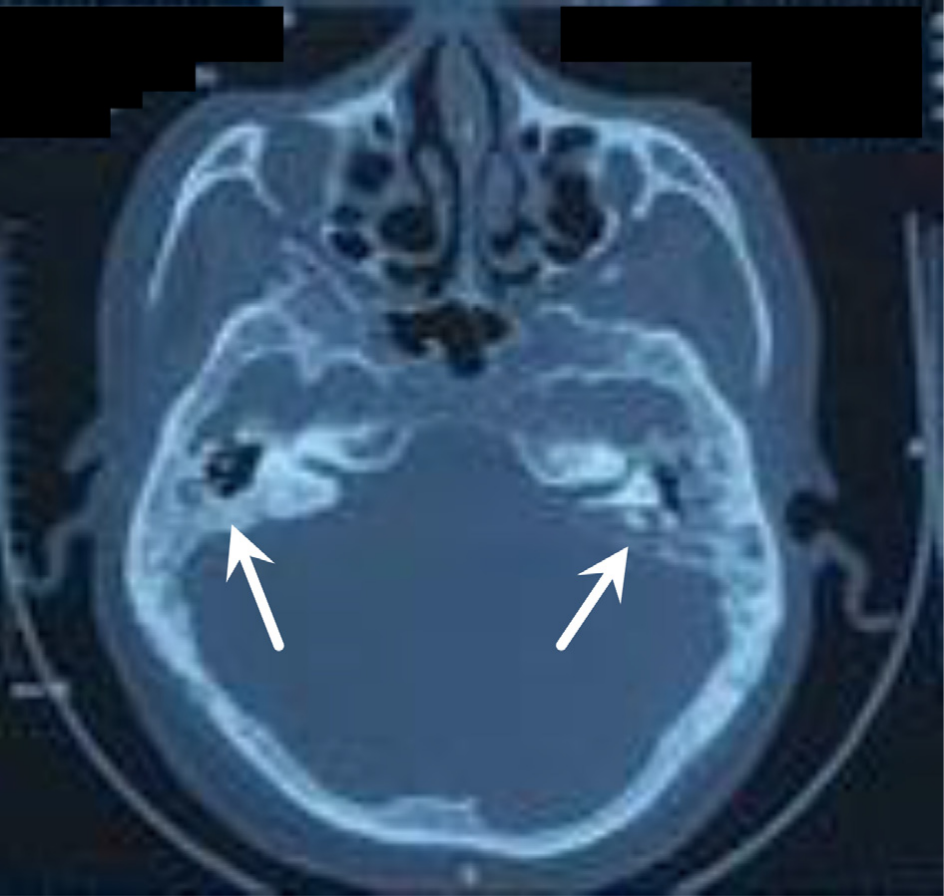
The MSCT of the head of the axial slice without contrast was sclerotic with left and right mastoid air-cell depletion (white arrow).

A man, 49 years old, came to the Emergency Unit (ER) RSUD Dr. Soetomo Surabaya with complaints of headaches for 5 months. The pain comes and goes and gets worse. A throbbing headache felt all over the head. For the past 4 months, the patient felt unbalanced walking, spinning, and never getting better. Complaints were accompanied by fever and vomiting. In the past year, the patient complained of ear pain accompanied by decreased hearing and a thick discharge from the right ear that he felt, but the patient did not go to the doctor. Physical examination revealed normal and conscious vital signs, with focal neurological deficits in the form of bidirectional nystagmus, positive open and closed Romberg test to the right, right past pointing dysmetria test, and right dysdiadokinesia. Complete blood count showed leukocytosis 15.980/uL, neutrophils 83.4%, C-reactive protein (CRP) 2.7 mL/dL, erythrocyte sedimentation rate (ESR) 30 mm/hour. The patient underwent a blood culture, but no bacterial culture was found. MRI examination showed multiple lesions, intra-axial, infratentorial, well-defined, regular edges, measuring about ±2.2 × 2 × 2 cm on the right cerebellum with perifocal edema, which was hypointense on T1-weighted, hyperintense T2-weighted on diffusion-weighted imaging (DWI). Contrast administration showed rim enhancement. The lesion was seen pressing the right posterior aspect of the pons and the fourth ventricle medially, accompanied by leptomeningeal enhancement and right mastoiditis (Fig. 1a). MR spectroscopy showed an increase in the perilesional lipid/lactate ratio. There was no increase in intra- or perilesional choline/creatin and choline/N-acetyl aspartate ratios (Fig. 2). On multislice computerized tomography (MSCT) examination, the head of the axial slice was sclerotic with decreased right and left mastoid air cells, which showed a picture of right and left mastoiditis (Fig. 3).

The patient was referred to the otolaryngology department and the dental and oral departments to find the source of the infection. The results of the examination revealed bilateral chronic suppurative otitis media. Then the patient underwent an audiometric examination and found that the sensorineural hearing loss was very severe, with pure tone audiometry (PTA) >120 dB. Dental and oral analysis revealed chronic apical periodontitis. The patient was given antibiotic therapy, namely ceftriaxone 2 g every 12 hours intravenously (IV) for 4 weeks and metronidazole 500 mg every 6 hours intravenously (IV) for 4 weeks. The patient had an MRI of the head with contrast 5 weeks after antibiotics administration. MRI of the head with contrast evaluation showed improvement. The size of the lesion appeared to be smaller, with a size of ±1.8 × 1.7 × 2.2 cm in the right cerebellar hemisphere (Fig. 1b).

The patient went to the outpatient clinic for clinical and radiological evaluation. Patient underwent an MRI of the head with contrast evaluation after administering metronidazole 500 mg every 6 hours (week 22) and cefixime 200 mg every 12 hours (week 13). MRI showed no restricted diffusion area lesions on DWI. The lesion showing contrast enhancement appeared to be shrinking with a size of ±0.6 × 0.6 × 0.9 cm in the right cerebellar hemisphere (Fig. 1c). Then the antibiotic cefixime was stopped, and metronidazole 500 mg every 6 hours was continued (28 weeks). The patient underwent a head MRI with contrast evaluation showing a relatively similar abscess size compared to week 22 (Fig. 1d).

## Discussion

Otogenic brain abscess is an accumulation of pus in the cerebrum or cerebellum that develops into encephalitis. Otogenic brain abscess is caused by pyogenic microorganisms originating from the inflammatory process in the middle ear cavity. Acute and chronic otitis media both cause autogenic brain abscesses. Possible pathways of propagation of brain abscess consist of direct extension pathway and hematogenous pathway. Among these, the most common cause is a direct extension of infection through the osteitic bone. Autogenic brain abscess usually presents as a solitary abscess rather than in multiple forms. The infection then develops in the temporal lobe 2-4 times more often than in the cerebellum. But there are many different reports about the location of the abscess. After the development of antibiotics, the incidence of autogenic brain abscesses decreased statistically. Although rare, the mortality rate is high enough to consider the possibility of intracranial complications in the treatment of otitis media. Symptoms of an otogenic brain abscess are generally mild in the early stages. Nonspecific symptoms such as headache, nausea, vomiting, and fever may develop in the early stages of a brain abscess. Danger signs/red flags that need attention in cerebellar abscess cases are headache, mental status changes, fever, and focal neurological deficits. Headache is the most common symptom of otogenic brain abscess in about 70%. Alterations in mental status (65%) and seizures (25%-35%) may occur due to meningeal irritation and increased intracranial pressure. In rare cases, lateralization may occur on motor examination. Nausea and vomiting occur in about 40%. Other neurological deficits are the stiff neck (40%) and papilledema (25%). Vertigo symptoms will be more dominant in the vestibulocerebellum structures (flocculus, paraflocculus, tonsils, and nodules). In addition, gaze-evoked nystagmus and saccadic movements are often found on physical examination, symptoms of head-shaking nystagmus appear (2%-15%), and central-positional nystagmus (12%-28%). The diagnosis of a cerebellar abscess in this patient was made based on anamnesis with complaints of chronic headache, fever, history of ear infection and focal neurological findings in the form of bidirectional nystagmus, positive open and closed Romberg test for right eye, right past pointing dysmetria test, right rebound phenomenon and right dysdiadokinesia [2].

Investigations in patients with brain abscesses can provide a general picture of infection, such as erythrocyte sedimentation rate, blood leukocytosis or elevated CRP. However, the infection blood parameters were in the normal range at 30%-40% [3]. In the case report, complete blood count showed leukocytosis 15,980 /uL, neutrophils 83.4%, C-reactive protein (CRP) 2.7 mL/dL, and erythrocyte sedimentation rate (ESR) 30 mm/hour, suggesting an infection. Brain imaging should be repeated to see the response to treatment in 1-2 weeks. If antibiotic therapy is adequate, the abscess will resolve with a gradual decrease in abscess size. Smaller abscesses may eventually heal with no residual abnormalities on imaging [4]. MRI examination provides higher sensitivity and specificity in the diagnosis of brain abscess. MR spectroscopy detects the products of bacterial metabolism (lactate, acetate, and succinate) and neutrophil proteolysis (cytosolic amino acids), which are used as support. It has even been reported that the combined use of DWI and MR spectroscopy increased the specificity and sensitivity to 95.2% and 100%, respectively [5,6].

Arlotti et al. stated that surgery should still be considered if antibiotic therapy fails after 1-2 weeks [7]. The study of Chowdhury et al. showed resolution of brain abscess after surgery followed by antibiotics in 162 cases with complete recovery of neurologic deficit in 80.86% of cases [8]. The study of Ndubuisi et al. showed no mortality in 11 cases of brain abscess undergoing conservative therapy related to the level of consciousness when the patient came [9]. Our patient is considering antibiotic therapy by closely monitoring the long-term antibiotic clinical condition and side effects. The choice of empiric treatment for brain abscesses should be guided by data on local antimicrobial susceptibility, source of infection, and immune status. A particular consideration in the treatment of brain abscesses is the ability of antimicrobials to penetrate the abscess and the CNS [4].

Before mass expansion occurs, early antibiotic therapy can prevent progression from cerebritis to abscess. Patients with symptoms for less than one week respond better to medical treatment than those who persist for more than one week. In certain circumstances, brain abscesses can be treated without surgery. Small abscesses (<2.5 cm) and cerebritis may respond to antibiotics alone. Patients treated with medical therapy alone improved clinically before the CT scan showed improvement. CT scan and MRI will show a decrease in lesion size and edema and a reduction in the number of lesions. Improvements on CT scans generally show complete resolution in 1-11 months, although radiological abnormalities may persist for months after successful therapy [10].

The early stages of cerebritis can be managed in a shorter time, about 4-6 weeks. However, patients with encapsulated abscesses, tissue necrosis, uncontrolled abscess growth, multiple abscesses, lesions at vital sites, and immunocompromise require 6-8 weeks. It takes a long time for brain tissue to repair and close the abscess space. Initial antibiotics are administered intravenously, followed by 2-6 months of oral therapy. Penetration of antibiotics is poor across the blood-brain barrier, so the choice of antibiotics is limited and maximum doses are often required [10]. In this case report, the patient was in the encapsulation stage, so the patient was considered for long-term use of antibiotics.

Empirical antibiotic therapy should be based on the most likely underlying etiologic agent, source of primary infection and pathogenesis. Parenteral antibiotics are active against pathogens, penetrate the abscess fluid and the site of the underlying disease in adequate concentrations and are bactericidal. The combination of penicillin or a third-generation cephalosporin (cefotaxime or ceftriaxone) plus metronidazole is effective empiric therapy in most cases [10]. Community-acquired brain abscesses in adults, a combination of intravenous cefotaxime 8-12 g/day or ceftriaxone 4 g/day, and intravenous metronidazole 1.5 g/day is recommended in the majority of cases [11]. Aerobic bacteria that can cause autogenic brain abscess include Staphylococcus, Proteus sp, and Streptococcus pneumonia, while anaerobic bacteria Peptococcus, Peptostreptococcus, and Bacteroides sp. Streptococcus pneumoniae is susceptible to empiric antibiotics [12].

Abscess evacuation surgery without proper antibiotic coverage is less meaningful in handling. Brain abscesses are generally treated with antimicrobial therapy for a minimum of 4-8 weeks but longer for abscesses treated with medical therapy without surgical excision [4], [5], [6], [7], [8], [9], [10], [11], [12], [13]. In this case, the patient experienced successful 28 weeks of adequate antibiotic therapy as seen on a contrast-enhanced head MRI showing resolution of the abscess diameter and clinical improvement of vertigo in the patient.

## Conclusion

Long-term antibiotic treatment can be an alternative if surgery cannot be performed. It should be accompanied by close monitoring of the side effects of antibiotics. Initial empiric antibiotic therapy should be based on the expected etiologic agent, possible predisposing conditions, source of primary infection and suspected pathogenesis. The antibiotic selected must be able to penetrate the abscess fluid and the site of primary infection in adequate concentrations and be bactericidal. Surgery is still considered if there is no good clinical response during medical therapy.

## References

[1] Kwak MK, Chung JH, Lee SH, Park CW. (2014). A case of otogenic brain abscess causing loss of consciousness. Korean J Audiol.

[2] Zwergal A, Feil K, Schniepp R, Strupp M. (2020). Cerebellar dizziness and vertigo: etiologies, diagnostic assessment, and treatment. Semin Neurol.

[3] Brouwer MC, Van De Beek D. (2017). Epidemiology, diagnosis, and treatment of brain abscesses. Curr Opin Infect Dis.

[4] Felicia Chow M. (2018). Neuroinfectious Disease. Brain Spinal Epidural Abscess.

[5] Muccio Carmine Franco, Caranci Ferdinando, D'Arco Felice, Cerase Alfonso, Lipsis Luca De, Esposito Gennaro, Tedeschi Enrico, Andreula Cosma (2014). Magnetic resonance features of pyogenic brain abscesses and differential diagnosis using morphological and functional imaging studies: a pictorial essay. J Neuroradiol.

[6] Sato J, Kuroshima T, Wada M (2016). Use of FDG-PET to detect a chronic odontogenic infection as a possible source of the brain abscess. Odontology.

[7] Arlotti M, Grossi P, Pea F (2010). Consensus document on controversial issues for the treatment of infections of the central nervous system: bacterial brain abscesses. Int J Infect Dis.

[8] Chowdhury F, Haque M, Sarkar M, Noman Khaled Chowdhury S, Hossain Z, Ranjan S (2015). Brain abscess: surgical experiences of 162 cases. Neuroimmunol Neuroinflammation.

[9] Shah A, Choudhri O, Jung H, Li G. Preoperative endovascular embolization of meningiomas: update on therapeutic options. 2015;38(March):1-8. doi:10.3171/2014.12.FOCUS14728.Disclosure. 25727229

[10] Brook I. (2017). Microbiology and treatment of brain abscess. J Clin Neurosci.

[11] Cantiera M, Tattevin P, Sonneville R. (2019). Brain abscess in immunocompetent adult patients. Rev Neurol (Paris).

[12] Cho SH, Park MK, Lee JD, Hwang SC. (2012). Otogenic brain abscess presenting with gait ataxia. Korean J Audiol.

[13] Chen M, Low DCY, Low SYY, Muzumdar D, Seow WT. (2018). Management of brain abscesses: where are we now?. Child's Nerv Syst.

